# A comparison of the Ranging behaviour and habitat use of the Ethiopian hedgehog (*Paraechinus aethiopicus*) in Qatar with hedgehog taxa from temperate environments

**DOI:** 10.1038/s41598-018-36117-5

**Published:** 2018-12-12

**Authors:** Carly E. Pettett, Afra Al-Hajri, Hayat Al-Jabiry, David W. Macdonald, Nobuyuki Yamaguchi

**Affiliations:** 1 WildCRU, Department of Zoology, University of Oxford, The Recanati-Kaplan Centre, Tubney House, Abingdon Road, Tubney, Abingdon, OX13 5QL UK; 2 Department of Biological and Environmental Sciences, College of Arts and Sciences, Qatar University, PO Box 2713, Doha, Qatar

## Abstract

We investigated seasonal changes in the ecology and behaviour of the Ethiopian hedgehog (*Paraechinus aethiopicus*) in Qatar, particularly in respect to differences in behaviour between hedgehogs living in arid environments and hedgehogs in temperate mesic environments. These comparisons will allow us to explore behavioural adaptations to different environments across hedgehog taxa. We radio-tracked 30 hedgehogs in Qatar over two years, and measured home range size, habitat preference, travel speed, activity and body mass. Whilst we found no difference in body mass between males and females, male home range size was over twice as large as that for females. Unlike hedgehogs in Europe, males maintained large home ranges during the non-breeding season. This behaviour may be sustained by the low cost of maintaining a large home range; males travelled less far per hour during the non-breeding season. Habitat use was non-random; arid areas with human influence, including rubbish dumping sites, was the most selected habitat type compared with its availability. Dense scrub and/or trees was the most selected habitat for nesting. This study gives us greater understanding as to how hedgehog taxa are adapted to their environment and therefore how they may be conserved, for example, the recent increase of “lower” level human activities, including irrigated farms and food waste, in harsh arid environments may have influenced the space use by Ethiopian hedgehogs.

## Introduction

The spatial pattern of animals is strongly influenced by the availability of resources including food, space, and potential mates^1–3^. Therefore, habitat use and ranging behaviour can vary both within species and within closely related species, depending on biotic conditions^4–7^. To understand the general behaviour and ecology of a group of organisms it is essential to study multiple taxa within the group, including those living in different environments. Although there are 16 described species of spiny hedgehog in the subfamily Erinaceinae^8,9^, the scientific literature mainly focuses on hedgehogs in the genus Erinaceus of temperate Europe. For example, the ranging behaviour and habitat use of the western European hedgehog (*Erinaceus europaeus*) is well studied across much of its range in western Europe, the UK, Ireland and Scandinavia^10–15^. Much less is known of hedgehog species inhabiting arid environments, such as desert hedgehogs (the genus Paraechinus), which are found from North Africa across the Middle East to India^8^.

The most widespread hedgehog in the genus Paraechinus is the Ethiopian hedgehog (*Paraechinus aethiopicus*), which is distributed across the Sahara, Middle East and the Arabian Peninsula, including Qatar^8,16^. The species is the only native hedgehog in Qatar^17^ and previous studies of the Ethiopian hedgehog in this country have shown that, akin to its European counterparts, the species is nocturnal^18^ and despite the warmer climate than in Europe, the species also enters hibernation/torpor during winter^18^. Breeding begins shortly after emergence from torpor^19^ and males have larger home ranges than do females during the breeding season^20^. It has been also reported that the species selects vegetated areas, such as oases and rawdhats^17,20^. Minimal data exist on the seasonal changes in ranging behaviour and habitat requirements of the species.

This study aims to investigate seasonal changes in the ecology and behaviour of the Ethiopian hedgehog by radio-tracking free-ranging individuals in Qatar, in order to examine intraspecific differences in spatial patterns as a result of biotic conditions. Firstly, theory dictates that individuals inhabiting less productive habitats will have larger home ranges^3,21,22^ and we hypothesise that Ethiopian hedgehogs will have larger home ranges than European hedgehogs living in mesic environments, and also hedgehog taxa living in semi-mesic environments. Secondly, water availability in arid environments is also a key driver of ranging behaviour and habitat use in small mammals^23,24^. Therefore, we also predict that water availability will be a key driver of habitat use for the Ethiopian hedgehog. Thirdly, the cost of thermoregulation in winter is expected to be lower in hotter climate^8^ and we predict differences in activity patterns in winter will be detectable between Ethiopian hedgehogs and hedgehogs living in cooler areas. These comparisons will allow us to explore behavioural adaptations to different environments across hedgehog taxa and help us to understand how this species can persist in challenging conditions, which has implications for its conservation.

## Results

### Ranging behaviour

We tracked 30 adult hedgehogs, 15 males and 15 females over a total of 178 tracking nights. The mean number of fixes per hedgehog over the study season was 381 ± 35 (range 65–758). To analyse differences in home range sizes we included 68 ranges from these 30 hedgehogs, 31 from the breeding season and 37 from the non-breeding season. We obtained 9775 measures of distance between hourly tracking fixes from the 30 hedgehogs.

**Figure 1:**
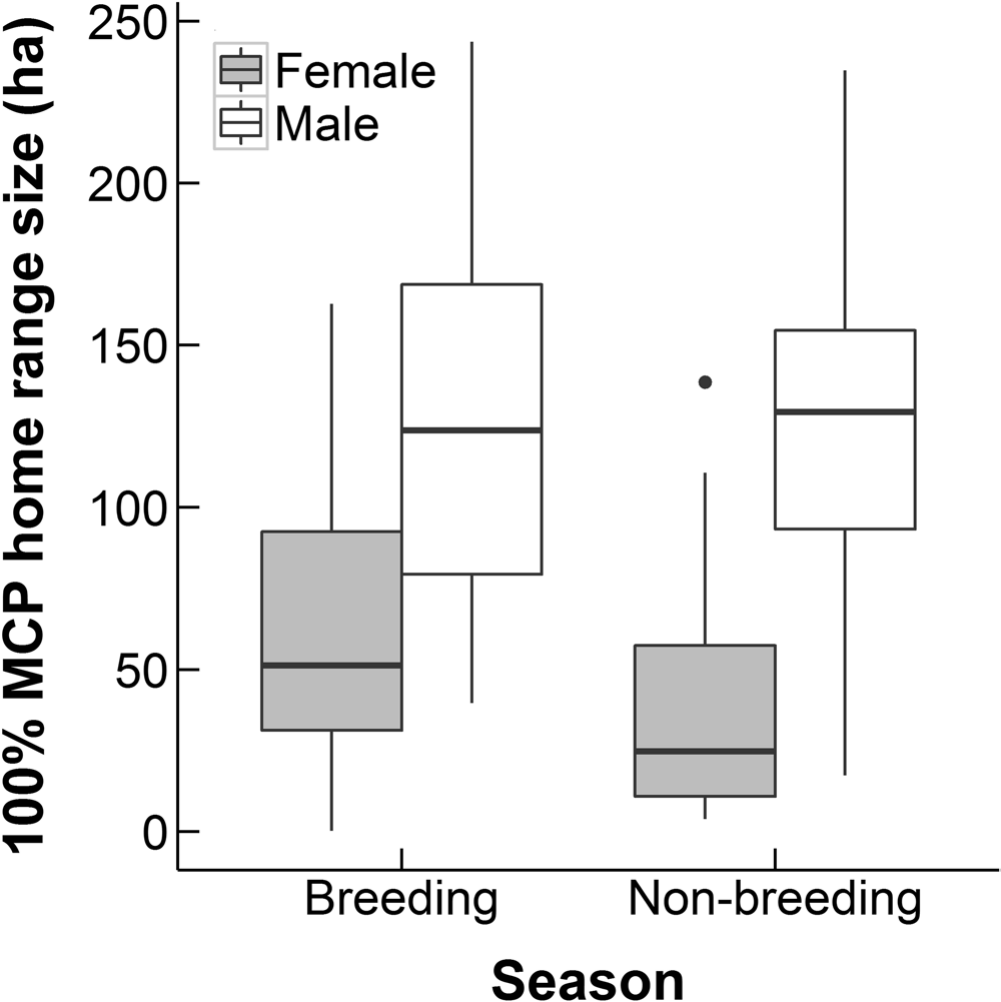
Sexual and seasonal variation in home range size, calculated by Minimum Convex Polygons (MCPs), for 68 Ethiopian hedgehogs.

The mean male home range size was over twice as large as the mean female home range size and the difference was statistically significant (100% MCP males split by year and season: 125.87 ± 10.19 ha, females: 53.30 ± 9.69 ha; Table 1, Figs 1, S1). We also found males had a significantly larger home range than females when ranges were calculated using 95% MCPs, whilst the difference was not significant when ranges were calculated using 50% Kernels (Table 1). We found no evidence to suggest an effect of year or season on home range size (Table 1), meaning that both males and females keep a constant home range size throughout the year.

**Table 1:** The results of a series of linear models to test for seasonal, annual and sex differences in hedgehog home range size measured by 100% and 95% Minimum convex polygons (MCPs) and 50% Kernels.

	Variable	Df	F	P value	Effect size	95% CI
100% MCP	(Intercept)				7.674	5.962, 9.376
	Sex *MALE*	1,26	17.578	<0.0001***	4.213	2.249, 6.144^†^
	Season *NON-BREEDING*	1,49	0.996	0.318	−0.638	−1.921, 0.584
	Year 2011	2,55	3.781	0.151	−0.309	−1.827, 1.143
	2012				−1.803	−3.704, 0.081
95% MCP	(Intercept)				5.615	3.821, 7.376
	Sex *MALE*	1,24	11.760	0.0006**	3.772	1.611, 5.894^†^
	Season *NON-BREEDING*	1,44	0.260	0.610	0.296	−0.893, 1.408
	Year 2011	2,49	1.680	0.432	−0.177	−1.602, 1.159
	2012				−1.128	−2.999, 0.694
50% Kernel	(Intercept)				3.441	2.042, 4.798
	Sex *MALE*	1,24	3.429	0.064.	1.644	−0.108, 3.359^†^
	Season *NON-BREEDING*	1,43	0.782	0.377	−0.340	−1.132, 0.399
	Year 2011	2,47	3.528	0.171	0.616	−0.352, 1.516
	2012				1.207	−0.173, 2.503-

Words in italics following categorical variables indicate the reference category.

***Significant at p < 0.001 level, **Significant at p < 0.01 level, *significant at p < 0.05.near significance.

^†^95% confidence interval of the effect size does not contain zero.

Response variables were square root transformed.

Females, breeding season and 2010 were the reference categories.

**Figure 2:**
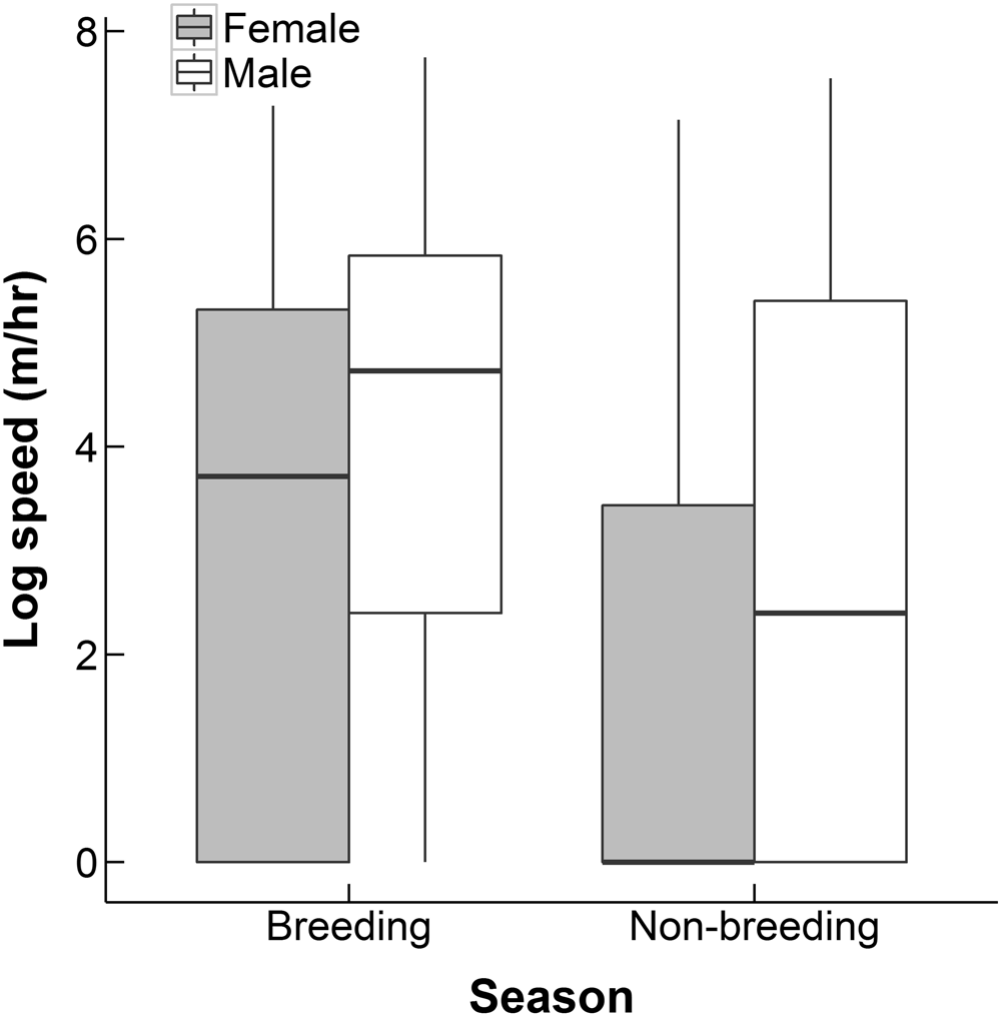
Sex and seasonal variation in the distance travelled by a hedgehog in one hour.

The mean distance travelled in one hour was 135.56 ± 2.38 meters, meaning hedgehogs travelled on average 1626.72 meters a night (over 12 hours). Male hedgehogs also travelled significantly faster (distance travelled per hour) than females (F_1,22_ = 19.13, p = 0.0002, Fig. 2). Males travelled a mean speed of 179.12 ± 3.72 meters an hour, compared with 83.17 ± 2.54 meters per hour for females. Speed varied significantly with season (F_1,1787_ = 64.67, p < 0.0001, Fig. 2). The distance travelled per hour was shorter in the non-breeding season for both males and females (Fig. 2). During the breeding season, the mean hourly speed was 195.78 ± 4.85 meters per hour and during the non-breeding season it was 103.42 ± 2.49 meters per hour. We found a positive correlation between travel speed and ambient temperature (F_1,9425_ = 478.58, p < 0.0001, Fig. S2), although it may be arguable if it is appropriate to describe this as a linear relationship. Travel speed was lower in 2010 than in the two following years (F_2,2924_ = 125.58, p < 0.0001).

When all years and seasons were combined home range size, measured by 100% MCP, ranged from 19.50 to 353.60 ha with a mean of 144.04 ± 16.95 ha. When broken down by year and season home range size ranged from 0.26 to 243.70 ha with a mean of 91.71 ± 8.29 ha.

### Body mass

**Figure 3:**
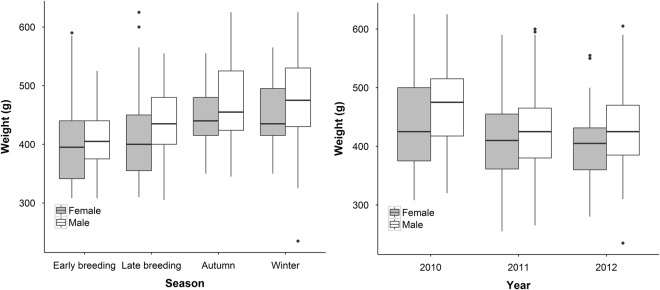
Left: seasonal and sex differences in the body mass of adult Ethiopian hedgehog. Right: annual differences in in the body mass of adult Ethiopian hedgehogs.

We obtained 1109 measures of body mass from 151 adult hedgehogs. We found no difference in body mass between males and females (F_3,131_ = 1.63, P = 0.2). The body mass of adult hedgehogs varied seasonally and was greatest in the autumn and winter seasons (F_3,902_ = 141.22, P < 0.0001, Fig. 3). Body mass did vary annually (F_2,940_ = 22.86, p < 0.0001); hedgehog body mass was greatest in 2010 (Fig. 3).

### Activity

**Figure 4:**
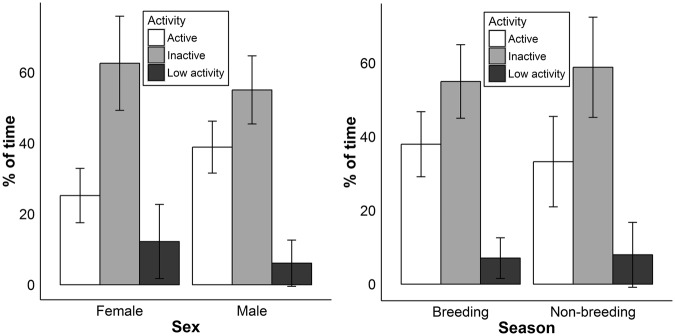
The mean percentage of time hedgehogs were deemed to be active, displaying low activity and inactive. Activity was determined from an activity sensor attached to a radio-tag. Bars indicate 95% confidence intervals of the means. Left: Sexual variation in activity. Right: Seasonal variation in activity.

When combining hedgehog radio-tracking fixes for all hedgehogs, they spent 37% their time active, 9% amount displaying low activity, and 54% inactive. We found that males spent a higher percentage of their time being active than females did, this was near statistical significance when activity was measured using activity sensors on the tag (F_1,42_ = 4.18, p = 0.076, Fig. 4) and significant when measured using the change of locations (F_1,42_ = 5.12, p = 0.022, Fig. S3). We found individuals spent a lower percentage of their time being active during the non-breeding season than in the breeding season, this was statistically highly significant when activity was measured using activity sensors on the tag (F_1,42_ = 193.66, p < 0.0001, Fig. 4) and when measured using the change of locations (F_1,42_ = 232.41, p < 0.0001, Fig. S3).

### Habitat Selection

**Figure 5:**
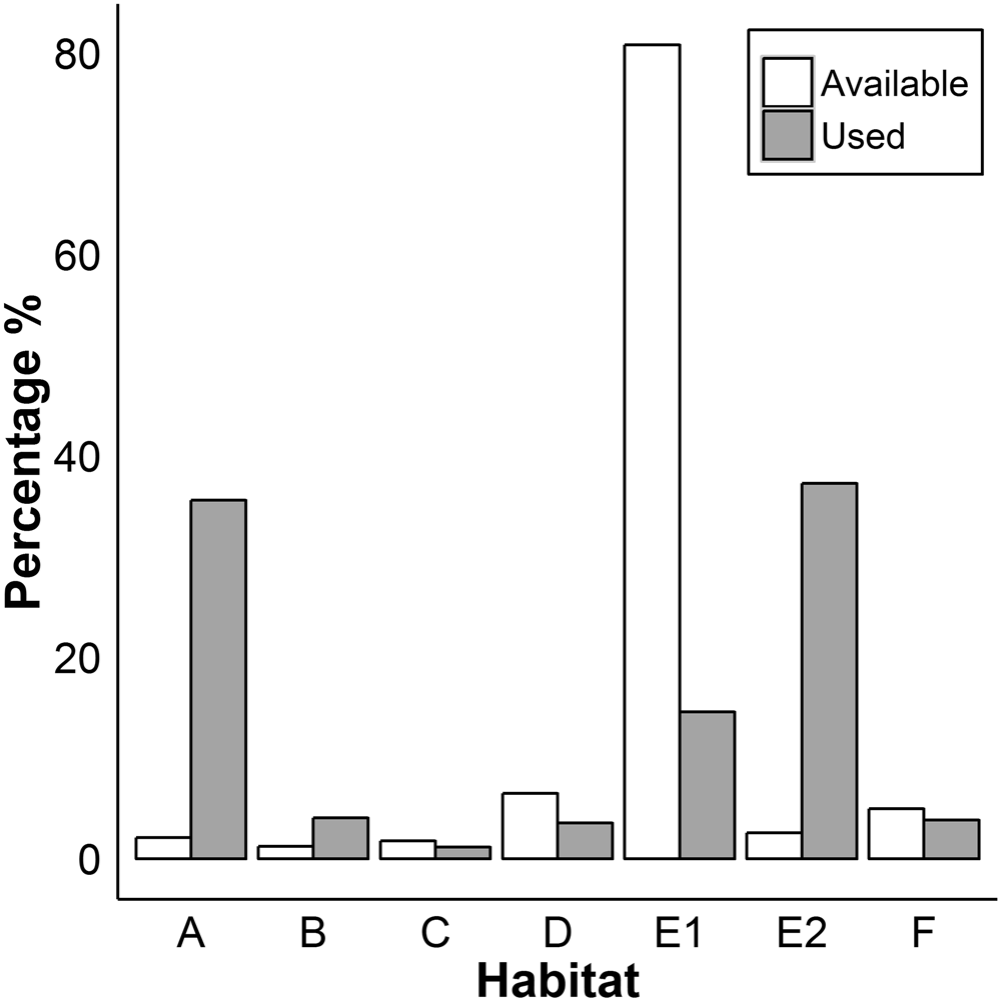
The percentage of each habitat available hedgehogs at a 15km^2^ study site in Qatar and the percentage of radio-tracking fixes in this habitat.

Habitat use was non-random and there were no differences in habitat rankings between seasons and sexes (Table 2, Fig. 5). For all individuals combined, both sexes and both seasons, habitat E2 (arid areas with direct human influence) was the most selected habitat compared with its availability. Habitat A (dense scrubs and/or trees) was the second highest ranked habitat. Habitat C (Plantations) was the lowest ranked habitat. There was a difference between compositional analysis performed on tracking fixes deemed to be from nesting hedgehogs and from all tracking fixes combined. For nesting habitat A was the most selected habitat compared with its availability, followed by E2. Arid areas with less human influence (E1) was the lowest ranked habitat for nesting (Table 2).

**Table 2:** The ranking of habitats selected by radio-tracked hedgehogs at a 15km^2^ site in Qatar.

Rank	6	5	4	3	2	1	0	p	λ
All	E2>	A>>>	B>	E1>	F>	D>	C	0.002*	0.041
Males	E2>	A>>>	B>	E1>	F>	D>	C	0.002*	0.026
Females	E2>	A>>>	B>	E1>>>	F>	D>	C	0.004*	0.040
Breeding	E2>	A>>>	B>	E1>	F>	D>>>	C	0.002*	0.110
Non-breeding	E2>>>	A>>>	E1>>>	B>>>	C>>>	D>	F	0.002*	0.045
Nests	A>	E2>>>	F>	B>>>	C>	D>>>	E1	0.002*	0.048

Habitats are ranked from most selected to least from left to right.

>>> indicates a statistically significant difference in hedgehog preference between habitat groups >indicates a non-significant difference.

Ranking was carried out following Aebischer, Robertson & Kenward 1993^36^.

**Figure 6:**
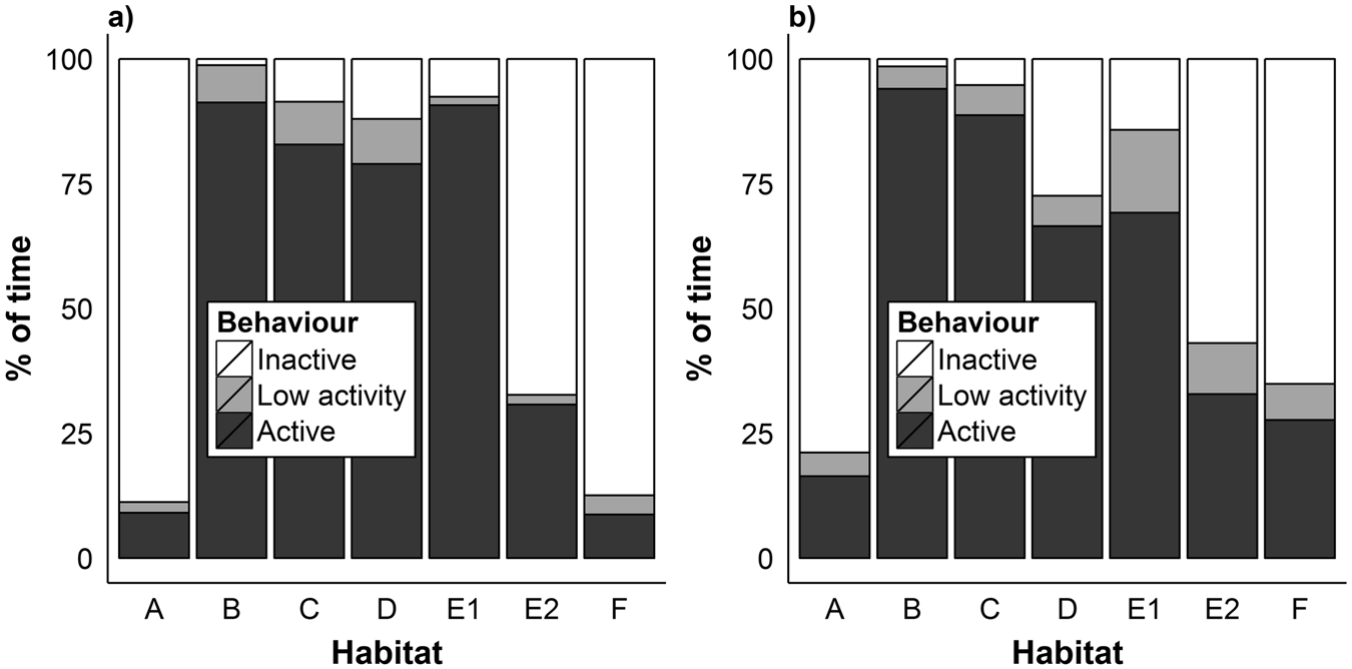
Variation in the observed activity level of hedgehogs in seven habitats in Qatar (**a**) activity level recorded from activity sensor attached to a radio-tag and (**b**) activity level recorded from change of locations. Habitats were as follows (A) dense scrubs and/or trees (usually irrigated), (B) regularly irrigated area without scrubs or trees, (C) plantations (e.g. date palm plantation), (D) open field on farmland, E1: arid areas with less human influence, E2: arid areas with direct human influence (e.g. dumping site), (F) built area (e.g. occupied houses).

Activity level varied between the habitats that the hedgehog was observed in, both when activity was measured by a sensor on the transmitter and by change of locations (Fig. 6). The pair-wise t tests corroborated the results for the compositional analysis (Table S1); hedgehogs spent more time inactive and less time active in habitat categories A, E2 and F than in other habitats. There were no significant differences in the amount of time displaying low activity between the habitats when measured by the sensor, yet when measured by the change of locations hedgehogs spent more time in low activity in habitat E2 than the other, and less time displaying low activity in habitat A than the other habitats (Table S1, Fig. 6).

## Discussion

### Sexual (But Not Seasonal) Difference in Home Range Size

As documented for Erinaceus species^8,25,26^, the Daurian hedgehog *Mesechinus dauuricus*^27^, and the long-eared hedgehog *Hemiechinus auritus*^28^, we found that males had larger home ranges than females, in spite of no sexual dimorphism being detected in body mass (Fig. 3). If body size – a metabolically based parameter – was the best predictor of home range size, clear sexual dimorphism in range size is not likely to occur in species where there is no sexual dimorphism in body mass, and other important factors (usually concerning reproduction) are likely influencing range size^29,30^. Studies in Sweden, Ireland, and Finland show male Erinaceus hedgehog ranges reducing post-breeding, and the sexual dimorphism in range size during the breeding season in Erinaceus is considered to be due to males ranging more widely than females to locate receptive females during the breeding season^8,14,25,26^. However in this study, male Ethiopian hedgehogs retained larger ranges than females even in the non-breeding season.

Based on available information it is difficult to speculate possible (reproductive) benefits for males to maintain large home ranges during non-breeding season. Concerning possible costs, however, our results show that males travel substantially more slowly during non-breeding season (Fig. 2), suggesting that males’ energy expenditure may not be as high as expected from the size of their ranges. Also because hedgehogs are not territorial^8^, maintaining a large home range is not associated with the cost of territorial defence. It is possible that males maintained larger home ranges because of a different response to dispersed resources in the study area than females. Relyea *et al*.^21^ found that male and female deer in a desert environment responded differently to habitat productivity, with males maintaining larger home ranges than females when productivity was low. It is possible that the male and female hedgehogs in this study have differing foraging strategies when habitat productivity is low. Further research is necessary on reproductive biology of the Ethiopian hedgehog to identify possible benefits of males ranging widely during non-breeding season.

We found that the home range size of Ethiopian hedgehogs was larger than both European hedgehog species; *Erinaceus europaeus* 6–47 ha in various locations in Europe^8^ and *Erinaceus concolor* 1–2 ha in Israel^28^, and also larger than recorded ranges of the long-eared hedgehog *Hemiechinus auritus* 2–5 ha in Israel^28^. Home range size was, however, smaller than that of the Daurian hedgehog *Mesechinus dauuricus*, which has home ranges of up to 2172ha^27^. The Daurian hedgehog has also be found to travel over twice as far over the course of a night, 3.38 km compared with 1.63 km in this study^31^. Zapletal *et al*.^27^ studied the Daurian hedgehog in the semi-arid steppes of Mongolia and conclude that hedgehogs in this dry environment may have to travel further to forage sufficiently. It is plausible that this low food resource level is also the reason behind the relatively large home ranges of the Ethiopian hedgehog.

### Seasonal (But Not Sexual) Difference in Body Mass

We found no difference in body mass between males and females, also found in studies of *Erinaceus europaeus*^8^. We found adult hedgehogs had a heavier body mass in the Autumn and Winter seasons. An increase in body mass before the onset of hibernation has frequently been observed in *Erinaceus europaeus*^32–34^ and is likely that the increase in body mass in the Autumn and Winter of this study was due to hedgehogs foraging more intensively to gain body fat to survive periods of torpor during the winter.

### Habitat Preferences

The compositional analysis of hedgehog habitat use demonstrated that habitat E2 (arid areas with direct human influence) was the most selected habitat compared with its availability and habitat A (dense scrubs and/or trees) was the second highest ranked habitat. The analysis of speed and activity levels in varying habitats at the study site indicated possible reasons for this habitat selection. Hedgehogs spent more time being active in habitats B, C, D, and E1. These habitats represent open or/and arid areas where we surmise that there is low level of resources, either food or shelter, or both. Thus hedgehogs may not stay in one place within such habitats for foraging, as either there is not much food or they expose themselves to potential predators (although there appear to be few predators hunting hedgehogs in Qatar currently^35^), and move through them quickly. Hedgehogs were more sedentary and less active in habitats A, E2 and F. Habitat A constituted dense scrubs and trees and we found that these areas were utilised by the animals as shade/cover during resting or day nesting sites. Shrubs have previously been found to be a key habitat for European hedgehogs^22,36,37^ and Daurian hedgehogs^31^. Habitat E2 included dumping sites which we observed to be a key food source for hedgehogs and thus hedgehogs would move slowly as they forage in this habitat where there were many hiding places (e.g. piles of various materials) as well. Also, we found that hedgehogs used habitat E2 (e.g. large piles of rocks and date palm leaves) as day nesting sites. Habitat F constituted built-up areas and is also an area with shelter and scavenging opportunities.

The analysis of hedgehog nesting showed that habitat A (dense scrubs and/or trees) was the most selected habitat for nesting relative to its availability. Studies of nesting in other species of hedgehogs have also shown that dense vegetation such as scrub, leaves, shrubs and foliage is the preferred nesting habitat^28,34,38,39^. In the high day temperatures of Qatar the shade offered by trees and scrub may be particularly important for thermoregulation of hedgehogs.

### Possible Anthropogenic Influence on Hedgehogs in Qatar

Habitats A, E2 and F are created by human activities in Qatar. It appears to be true that some human activities, such as building cities, large roads, large-scale modern agriculture, reduce hedgehog populations^8,37^. However, studies have shown that in Europe *Erinaceus europaeus* is attracted to built-up areas, possibly due to the availability of supplementary food in urban areas than on intensively managed farmland^15,40,41^ and because of the shelter buildings offer from predation^15,36,42^. Our results highlight that Ethiopian hedgehogs in Qatar may also take advantage of human altered habitats; the current level of human activities in the study site (especially creating irrigated farms) may benefit the local Ethiopian hedgehog population by increasing resources both in food and shelter.

Resource availability may also be a function of environmental conditions. In this study we found a positive correlation between travel speed and ambient temperature, which could be an indication of hedgehogs being more active when invertebrate prey species are more active, also observed in *Erinaceus europaeus* in the UK^33^. We also found some interesting annual differences in hedgehog ranging behaviour. Travel speed was lower in 2010 than in the two following years and body mass was greatest in 2010. These annual differences may be a reflection of differences in resource availability and distance travelled for foraging between the years of the study. If so, it may suggest that, if buildings and fences that hedgehogs cannot move through would increase, such structures may negatively influence hedgehog’s body condition as they likely have to travel faster/longer along those structures in comparison to the situation without such structures.

## Conclusions

In summary, this study has revealed that male home range size was over twice as large as female home range size, and males also travelled faster and spent a higher percentage of their time being active than females. However, unlike hedgehogs in Europe, males maintained larger home ranges than their energy requirements predict during non-breeding season. Although it is difficult to speculate the benefit to maintaining a large home range throughout the non-breeding season, we tentatively consider that the cost may not be very high, partially due to their substantially reduced travelling speed in non-breeding season. Additionally, males and females may have differing foraging strategies in response to dispersed resources. We also observed a relatively larger home range size in the Ethiopian hedgehog in comparison to temperate species, likely to be a result of the Ethiopian hedgehog’s adaption to the desert environment.

We found many similarities between the Ethiopian hedgehog and the Western European hedgehog, such as the selection of habitats altered by human influence, the selection of dense scrub for nesting, and an increase in body mass in Autumn and in Winter. The findings of this research highlight the importance of resources created by humans, including food waste and dense tress and scrubs in irrigated farms, for Ethiopian hedgehogs, which may be beneficial for hedgehogs to survive in the desert environment.

## Methods

### Study area

**Figure 7:**
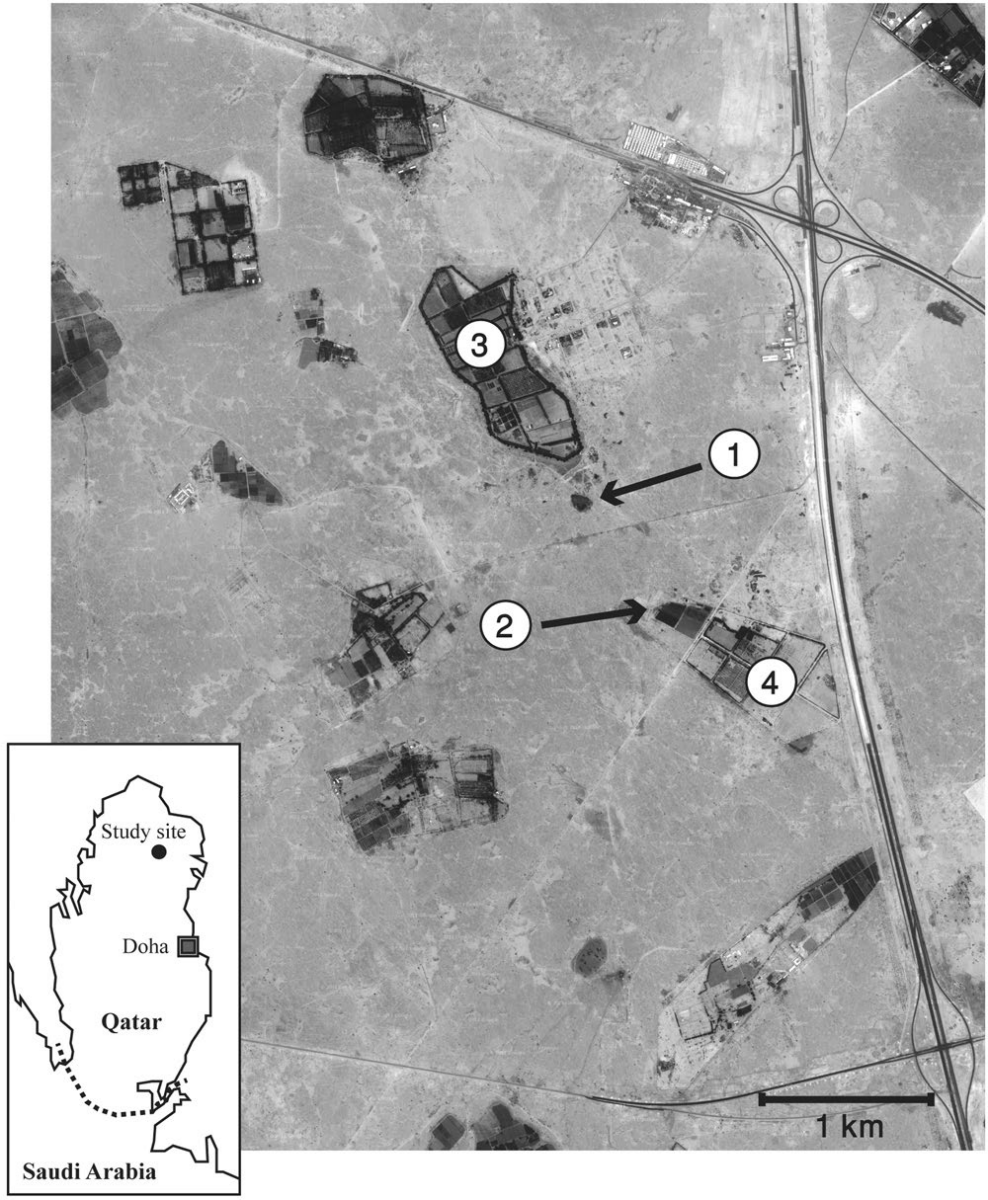
Map of the study site (GoogleEarth Image Copyright 2018 DigitalGlobe): (1) The “Rubbish Mound” where a higher concentration of hedgehogs was found throughout the year probably due to year-round availability of food resources. (2) “Municipal Farm” where permanent grass fields attracted hedgehogs. (3) Rawdat Al-Faras Research Station where street lights across the farm increased the chance of locating hedgehogs. (4)Qatar University Farm where the field station was located.

The study area consisted of c. 15km^2^ of arid land around the Qatar University Farm (25°48′ N, 51°20′ E) northern Qatar (Fig. 7). The area included 11 active farms which were irrigated daily. Except those farms, the area was an arid plain with the total annual precipitation of less than 100 mm, and the surface was predominantly covered by desert pavement with many exposed loose gravels. The ambient air temperature ranges between c. 5 °C in the early morning in winter and c. 50 °C in the early afternoon in summer. There was little vegetation except isolated short acacia tress and ephemeral grass patches emerging after rains in cooler months (usually between November and March). Various structures created by human activities, such as rubbish dumps, piles of abandoned building materials, and soil mounds, were ubiquitously found across the study area.

### Hedgehog capture

Hedgehogs were captured and tracked between April 2010 and April 2012. A consecutive four-day hedgehog survey was conducted once a month. Special attention was paid to 1) the “Rubbish Mound” where a higher concentration of hedgehogs was found throughout the year probably due to year-round availability of food resources, 2) “Municipal Farm” where permanent grass fields attracted hedgehogs, 3) Rawdat Al-Faras Research Station where street lights across the farm increased the chance of locating hedgehogs, and 4) Qatar University Farm where the field station was located (Fig. 7). In addition to captures at those sites, hedgehogs were captured, and their behaviours observed, wherever and whenever they were found in the study area. We recorded 1190 captures consisting of 179 different animals, including 87 males and 74 females. Hedgehogs were captured and handled on site without anaesthesia or sedation, sexed, weighed, and individually marked by painting the spine with nail polishes of different colours. A new individual was classified as a juvenile if it weighed less than 160 g, as an adult if it weighed more than 370 g, and a subadult if it weighed between 160 g and 370 g^19^. If the history of an animal was known through the capture survey, any individual which was considered older than six months (likely the age of sexual maturity^8^), or survived the first winter into the spring breeding peak (February–March^18,19^), was classified as an adult. However, this age classification based on body weight should be considered applicable only to our study site during study period as the body weight of Ethiopian hedgehogs changes substantially depending on food availability^43^. It was not feasible to take measurements of various body parts (e.g. total length) of free-ranging Ethiopian hedgehogs in the field without anaesthesia because animals curled up.

### Radio-tracking

Thirty captured adult animals were fitted with VHF radio-transmitters (TW51 single celled tag, 164 MHz frequency range, Biotrack Ltd., Wareham, UK), and followed between dusk and dawn using hand-held flexible three element Yagi aerials and Sika receivers (Biotrack). We made one radio-fix per hour per animal wherever possible. A preliminary observation had revealed that hedgehogs travelled c. 1.5 km within 15 min, suggesting they are capable of travelling between the two furthest points of their home ranges within 30 min (Yamaguchi unpublished). Therefore, we considered that location fixes with intervals of at least one hour would be sufficient to reduce (if not remove) autocorrelation bias^44^. We were usually able to accurately locate the animal from a close distance (e.g. less than 50 m) with minimum disturbance to the animal when it was in an area with many different physical features (e.g. dense vegetation, dumping site, pile of building materials). When an animal was in an open area it appeared to run away from approaching observers, and hence the radio-tracking was carried out by following standard triangulation methodology^45^, usually within c. 200 m from the focal animals. Locations were recorded as coordinates on the map to the nearest 10 m. Prior to radio-tracking, we evaluated the triangulation error. Triangulation at c. 100 m and c. 200 m ranges gave the average fixation error of 12 ± 2.9 m and 31 ± 5.7 m, respectively (Mean ± SE, N = 5 for each,).

During the radio-tracking, in addition to the focal animals’ locations, temperature, relative humidity, focal animal’s activity level (inactive, low activity, active) and habitat type where the focal animal was located were recorded. The activity level was classified as follows after listening to the signal from the transmitter for one minute. When a transmitter was equipped with an activity sensor (ACT, Biotrack), we classified an animal’s activity level as either inactive (only inactive signals), low activity (active signals <50%), or active (active signals >50%). When a transmitter was not equipped with the activity sensor, it was classified as either inactive (an animal was in its day nest), active (an animal was out of its nest), low activity (an animal was out of its nest, and was on the same location at more than two consecutive fixes) – low activity was given post-hoc, and needed to be confirmed by an observation and was rarer than were the other two categories. We defined day nest as the location where a focal animal spent during the day prior to radio-tracking.

The habitat use of a radio-tracked animal was recorded with one of the following seven habitat categories, which was the dominant habitat type within c. 5 m radius of the focal animal; A: dense scrubs and/or trees (usually found only around irrigation ditches), B: regularly irrigated area without scrubs or trees (e.g. irrigated grass field), C: plantations (e.g. date palm plantation), D: open field in the farm, E1: arid areas with less human influence (e.g. monotone flat desert with little vegetation), Habitat E2: arid areas with direct human influence (e.g. dumping sites, piles of materials such as rocks and date palm leaves, and soil mounds), and F: built area (e.g. occupied houses, roads, and construction sites). The habitats A, B, C, and D were found only within regularly-irrigated farms.

### Data analysis

For the analysis of home range sizes data were pooled into two hedgehog seasons, breeding; February–July and non-breeding; August–January. For the analysis of body mass we had sufficient hedgehog captures to split in to four hedgehog seasons as follows^19^; early breeding season: February–April, late breeding season: May–July, autumn season: August–October and winter season: November–January.

Home ranges were calculated using the package adehabitatHR in the R software (R Core Team 2014). We used 100% and 95% minimum convex polygons (MCP) to give a simplistic measure of home range size and because this method has been used in a large number of hedgehog home range studies^8^. The area of the core home range size was also calculated using 50% Kernels. Home ranges were calculated for each individual for each season (breeding and non-breeding) and year. According to Morris 1988^11^, based on his study on the western European hedgehog in the UK, it is desirable for a researcher to follow a hedgehog for at least six hours a night over more than seven consecutive nights to estimate the size of its home range. We included only individuals which were followed for more than six hours a night on more than 10 days into the analyses concerning home ranges. However, to increase the sample size, if cumulative curves would suggest appropriate, individuals with less than the foregoing criteria were also included in home range analyses. All home ranges included in analysis had over 30 tracking fixes as recommended by Kenward^45^. All statistical analyses were carried out in the R software (R Core Team 2014). Travelling speed was also calculated from the straight distance between two consecutive hourly fixes and a mixed-effects model constructed to test for seasonal, sexual, annual and temperature differences in speed, with animal ID as a random factor.

To analyse variation in activity levels we constructed a mixed effects model to test for seasonal and sexual differences in the proportion of time an individual was active with animal ID as a random factor. We ran this model for activity data both based on the activity sensor attached to the radio-tag and based on location changes by animals.

We digitised the habitats available to the hedgehogs at the field site and to investigate habitat selection we carried out compositional analysis^46^. We also performed compositional analysis on a subset of tracking data deemed to be when hedgehogs were in their day nests. To investigate relationships between activity levels and habitats the percentage of tracking fixes where a hedgehog was displaying a given activity level was calculated for each habitat and pair-wise t tests were performed with a Bonferroni correction.

We ran a further mixed effects model to test for seasonal, annual and sex differences in body mass. All results are given as mean ± standard error unless otherwise stated.

### Ethical Approval

The work was approved by the Qatar University Institutional Review Board (approval number: QUIRB 136-E/12). All experiments were performed in accordance with relevant guidelines and regulations.

## Electronic supplementary material

Supplementary Information

## Data Availability

After publication of all papers related to this data the authors are happy to provide the raw data on request.
