# Supplementary material for: A comparison of the Ranging behaviour and habitat use of the Ethiopian hedgehog (Paraechinus aethiopicus) in Qatar with hedgehog taxa from temperate environments
Source: Sci Rep. 2018 Dec 12;8:17783. doi: 10.1038/s41598-018-36117-5 (PMC6290762; doi:10.1038/s41598-018-36117-5)
Supplement: Supplementary file 1 — Supplementary Information [file 41598_2018_36117_MOESM1_ESM.docx]

**A comparison of the Ranging behaviour and habitat use of the Ethiopian hedgehog (*Paraechinus aethiopicus*) in Qatar with hedgehog taxa from temperate environments- *Supplementary Information***

Carly E. Pettett^1^, Afra Al-Hajri^2^, Hayat Al-Jabiri^2^, David W. Macdonald^1^ and Nobuyuki Yamaguchi^2^*

^1^WildCRU, Department of Zoology, University of Oxford, The Recanati-Kaplan Centre, Tubney House, Abingdon Road, Tubney, Abingdon OX13 5QL, UK

^2^Department of Biological and Environmental Sciences, College of Arts and Sciences, Qatar University, PO Box 2713, Doha, Qatar

* Corresponding author: Nobuyuki Yamaguchi (email: yamaguchi@qu.edu.qa)

**Table S1** The results of pair-wise t tests to test for differences between the percentages of time spent displaying a level of activity in differing habitats. Left: activity level behaviour recorded from activity sensor attached to a radio-tag and right: activity level recorded from change of locations

| Activity levels from sensor | | | | | | |
| --- | --- | --- | --- | --- | --- | --- |
|  | Inactive | | | | | |
|  | A | B | C | D | E1 | E2 |
| B | **0.000** | - | - | - | - | - |
| C | **0.000** | 1.000 | - | - | - | - |
| D | **0.000** | 1.000 | 1.000 | - | - | - |
| E1 | **0.000** | 1.000 | 1.000 | 1.000 | - | - |
| E2 | 1.000 | **0.000** | **0.000** | **0.000** | **0.000** | - |
| F | **0.000** | 1.000 | 1.000 | 1.000 | 1.000 | **0.001** |
|  | Low activity | | | | | |
|  | A | B | C | D | E1 | E2 |
| B | 1.000 | - | - | - | - | - |
| C | 0.640 | 1.000 | - | - | - | - |
| D | 0.620 | 1.000 | 1.000 | - | - | - |
| E1 | 0.710 | 1.000 | 1.000 | 1.000 | - | - |
| E2 | 1.000 | 1.000 | 0.870 | 0.420 | 0.460 | - |
| F | 1.000 | 1.000 | 1.000 | 1.000 | 1.000 | 1.000 |
| Active | | | | | | |
|  | A | B | C | D | E1 | E2 |
| B | 0.068 | - | - | - | - | - |
| C | **0.000** | 0.942 | - | - | - | - |
| D | 0.277 | 1.000 | 0.100 | - | - | - |
| E1 | 1.000 | **0.016** | **0.000** | 0.072 | - | - |
| E2 | 1.000 | **0.013** | **0.000** | 0.078 | 1.000 | - |
| F | **0.007** | 1.000 | 1.000 | 0.410 | **0.002** | **0.002** |

| Activity from radio-tracking signal | | | | | | |
| --- | --- | --- | --- | --- | --- | --- |
|  | Inactive | | | | | |
|  | A | B | C | D | E1 | E2 |
| B | **0.000** | - | - | - | - | - |
| C | **0.000** | 1.000 | - | - | - | - |
| D | **0.000** | 0.678 | 0.623 | - | - | - |
| E1 | **0.000** | **0.041** | **0.043** | 1.000 | - | - |
| E2 | 1.000 | **0.000** | **0.000** | **0.000** | **0.000** | - |
| F | **0.000** | 0.632 | 0.591 | 1.000 | 1.000 | **0.000** |
|  | Low activity | | | | | |
|  | A | B | C | D | E1 | E2 |
| B | **0.002** | - | - | - | - | - |
| C | **0.001** | 1.000 | - | - | - | - |
| D | **0.003** | 1.000 | 1.000 | - | - | - |
| E1 | 1.000 | **0.026** | **0.013** | **0.022** | - | - |
| E2 | 1.000 | **0.004** | **0.002** | **0.002** | 1.000 | - |
| F | **0.037** | 1.000 | 1.000 | 1.000 | 0.141 | **0.016** |
| Active | | | | | | |
|  | A | B | C | D | E1 | E2 |
| B | 0.384 | - | - | - | - | - |
| C | **0.000** | 0.070 | - | - | - | - |
| D | **0.000** | 1.000 | 0.190 | - | - | - |
| E1 | 1.000 | **0.020** | **0.000** | **0.000** | - | - |
| E2 | 1.000 | 0.964 | **0.001** | **0.022** | 1.000 | - |
| F | **0.000** | 0.062 | 1.000 | 0.471 | **0.000** | **0.001** |

Habitats were as follows A: dense scrubs and/or trees (usually irrigated), B: regularly irrigated area without scrubs or trees, C: plantations (e.g. date palm plantation), D: open field on farmland, E1: arid areas with less human influence, E2: arid areas with direct human influence (e.g. dumping site), F: built area (e.g. occupied houses)

Percentages were arc sin transformed prior to analyses.

Bonferroni correction was applied

**
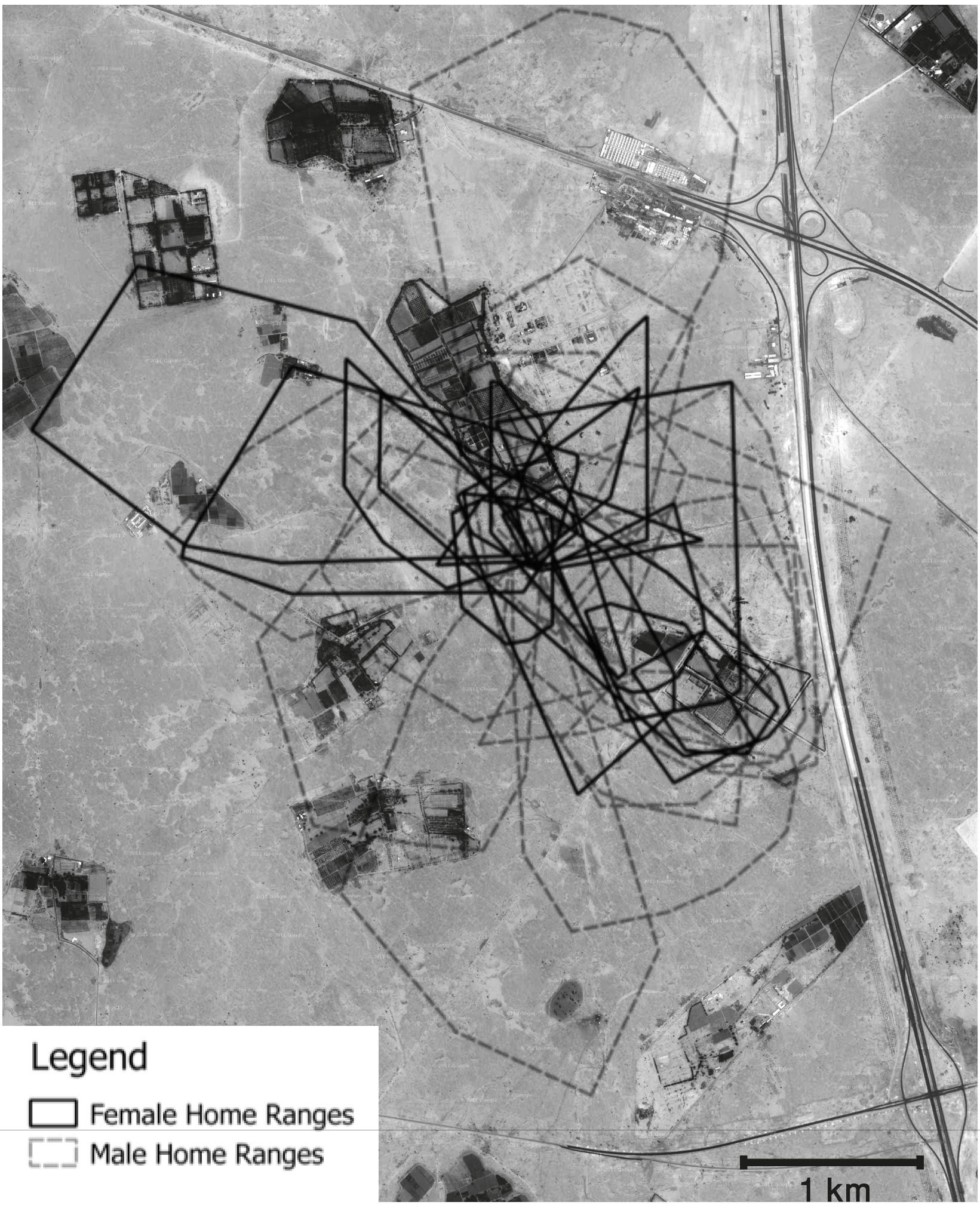
**

**Fig.S1** The home ranges of all of the Ethiopian hedgehogs tracked in the study, including tracking fixes from all seasons and years of the study (Satellite imagery: GoogleEarth Image Copyright 2018 DigitalGlobe, home ranges drawn in QGIS: QGIS Development Team (2018). QGIS Geographic Information System. Open Source Geospatial Foundation Project. http://qgis.osgeo.org)


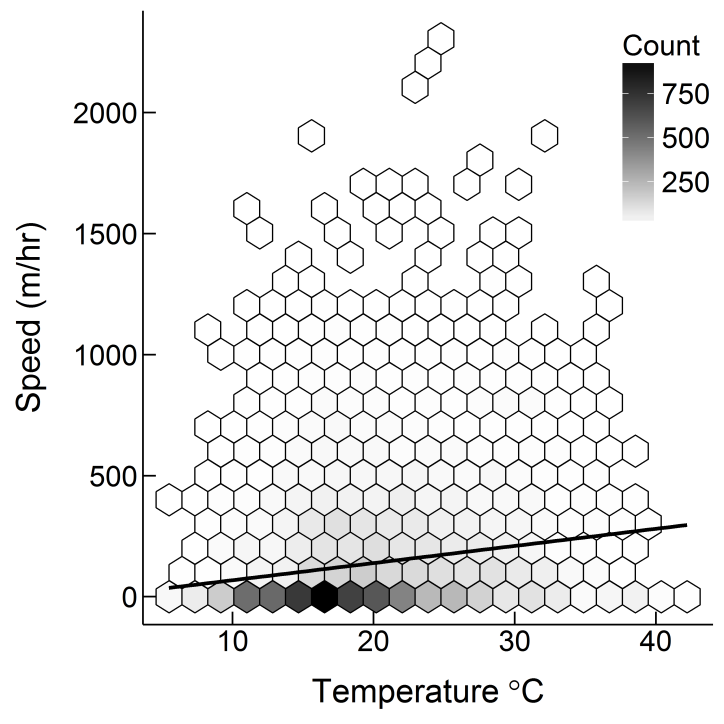
**Fig.S2** The relationship between ambient temperature and the distance travelled by a hedgehog in one hour


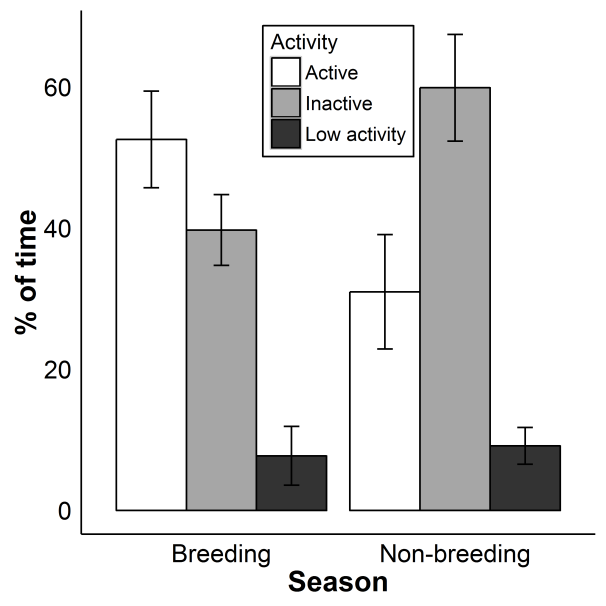

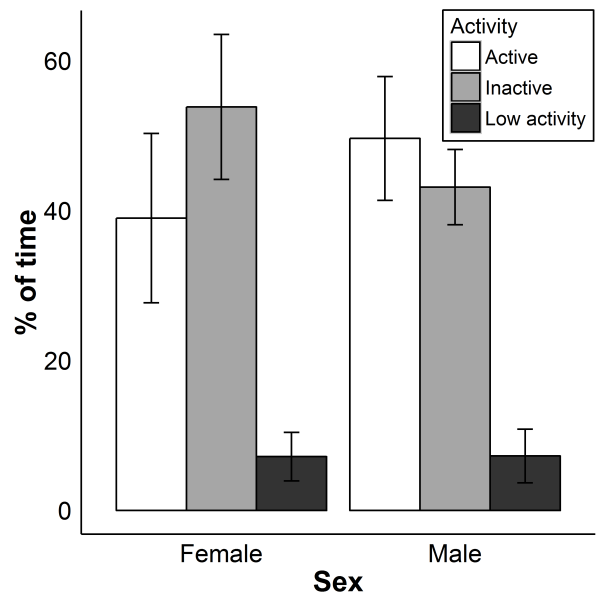


**Fig.S3** The mean percentage of time hedgehogs were deemed to be active, displaying low activity and inactive. Activity was determined from using the change of radio-tracking locations. Bars indicate 95% confidence intervals of means. Left: Sexual variation in activity. Right: Seasonal variation in activity
